# Reply to Cangelosi, G. Comment on “Inácio et al. Nursing Practice Environment in the Armed Forces: Scoping Review. *Nurs. Rep.* 2025, *15*, 394”

**DOI:** 10.3390/nursrep16040118

**Published:** 2026-04-01

**Authors:** Mafalda Inácio, Maria Carvalho, Ana Paulino, Patrícia Costa, Ana Rita Figueiredo, Elisabete Nunes, Paulo Cruchinho, Pedro Lucas

**Affiliations:** 1 Nursing Research Innovation and Development Centre of Lisbon (CIDNUR), School of Nursing, Universidade de Lisboa, 1900-160 Lisbon, Portugalpjcruchinho@enfermagem.ulisboa.pt (P.C.);; 2 Nursing Administration Department, School of Nursing, Universidade de Lisboa, 1900-160 Lisbon, Portugal; 3 Serviço de Cirurgia Geral e Gastrenterologia, Instituto Português de Oncologia de Lisboa Francisco Gentil, 1099-023 Lisboa, Portugal

We would like to thank the author of the comment on our article [1], “Nursing Practice Environment in the Armed Forces: Scoping Review”. We are grateful for the author’s recognition of our work and the acknowledgment of its potential contribution to the scientific community. Also, we sincerely thank the author for the generous appraisal of our scoping review and for noting that “the work stands out for its solid methodological rigor.” We appreciate this recognition, as the review was developed in accordance with the Joanna Briggs Institute methodology and reported in line with the PRISMA-ScR guidance.

As stated in the comment, the manuscript provides “a comprehensive analysis of the nursing practice environment in military contexts,” emphasizing leadership, collaboration, professional development, and resource adequacy as key determinants of nurse well-being, retention, and patient safety. We are grateful for this acknowledgement and for the author’s effort to expand the discussion, particularly by emphasizing the potential applicability of these dynamics across different organizational and operational contexts.

## 1. Nursing Well-Being and Burnout Prevention

We appreciate the author’s reflections on the relationship between the nursing practice environment and nurse well-being, particularly regarding burnout prevention and workforce sustainability. The comment emphasizes that, as demonstrated in our review, the nursing practice environment constitutes “a central dimension in relation to nurse well-being, burnout, and staff retention.” We find particularly valuable the author’s effort to extend this discussion to high-complexity civilian care settings, such as emergency services and correctional healthcare. This perspective aligns with research demonstrating that unfavourable work environments, characterized by inadequate staffing, excessive workloads, limited participation in decision-making, insufficient leadership and organizational support, poor interprofessional collaboration, and high emotional demands [2,3,4,5], are consistently associated with increased burnout, psychological distress, and higher turnover intentions among nurses [6,7,8,9,10]. Indeed, the literature increasingly recognizes the nursing work environment as a key structural determinant of both professional well-being and patient outcomes [11,12,13,14,15,16,17].

Studies have shown that supportive organizational environments, effective leadership, and adequate resources can mitigate burnout and improve job satisfaction, contributing to safer and higher-quality care [18,19,20,21,22,23,24,25]. The author’s observation that these dynamics are observable across diverse healthcare contexts further reinforces the understanding that the quality of the work environment represents a cross-cutting determinant of professional well-being in nursing.

## 2. Nursing Leadership and Care Management in Complex Setting

We also appreciate the author’s thoughtful reflections regarding the role of leadership in shaping nursing practice environments, particularly in complex organizational settings. This is consistent with research demonstrating that leadership behaviours are among the most influential determinants of nursing work environments and professional outcomes [22,23,24,26,27,28,29].

The author’s reference to complex civilian healthcare contexts, particularly in relation to the care of migrant populations, provides a valuable extension of the discussion presented in our review. The challenges associated with cross-cultural care appropriately illustrate how nursing leadership competencies (such as communication, negotiation, and empowerment) are essential for managing complex relational dynamics within diverse healthcare environments [30,31].

This perspective reinforces the understanding that leadership in nursing is not limited to administrative coordination but also involves the capacity to navigate cultural diversity [30,31,32]. The literature on transcultural nursing similarly emphasizes that culturally competent leadership and effective communication are critical for ensuring equitable and safe care for migrant and culturally diverse populations [32]. In these contexts, nurse leaders play a key role in fostering inclusive team environments, supporting culturally responsive care practices, and strengthening team resilience when professionals face unfamiliar cultural, linguistic, and social challenges [30,31,32,33,34]. We appreciate the author’s comparison and the identification of this important parallel between military healthcare environments and complex civilian care settings: both require adaptive leadership capable of integrating relational, cultural, and organizational competencies to sustain effective care delivery.

## 3. Resilience in Crisis Contexts

We also appreciate the author’s reflections on resilience within crisis contexts, particularly the emphasis on how organizational support, clear role definition, and interprofessional collaboration contribute to the psychological well-being of healthcare professionals working in demanding environments. The author’s observation that resilience should not be viewed solely as an individual attribute but rather as the result of supportive organizational environments is particularly important. The literature increasingly conceptualizes resilience in healthcare as a collective and organizational capability that emerges from supportive leadership, effective teamwork, and adequate structural resources [29,35,36,37]. In this sense, resilient healthcare organizations are those that foster clear communication, collaborative cultures, and adequate staffing, enabling professionals to maintain performance and well-being even under conditions of uncertainty and crisis.

## 4. Practical Implications and Future Directions

We also appreciate the author’s reflections regarding the practical implications of improving the nursing practice environment and the importance of developing strategies that support nurses working in complex healthcare contexts. The topics presented by the author are particularly insightful, as they further enrich and complement the discussion presented in our review. The comment outlines several key approaches, including adaptive leadership, interprofessional collaboration, adequate staffing, and professional development, which are widely recognized as essential components of healthy nursing work environments [2,13,14,17,38,39]. Finally, we agree with the author that continued research in this field is essential. Expanding empirical studies across different healthcare systems and cultural contexts may further clarify how leadership, organizational support, and professional development initiatives contribute to resilient nursing teams and sustainable healthcare organizations. Such efforts may help inform evidence-based strategies for improving nursing practice environments.

## 5. Conclusions

We appreciate the author’s concluding reflections emphasizing the importance of the nursing practice environment as a key determinant of nurse well-being, retention, and patient safety. As the author suggests, recognizing the parallelism between emerging military and civilian evidence may contribute to the development of sustainable strategies capable of reinforcing healthcare systems facing increasingly complex and global challenges. In this context, nurse managers play a central role in shaping practice environments, guiding organizational processes, supporting team dynamics, and promoting safe and high-quality patient outcomes.

Once again, we would like to thank the author for the thoughtful engagement with our work. It was with great interest and appreciation that we read the comment and prepared this response, as it provided an opportunity to further deepen and expand the topics addressed in our review. We consider this exchange constructive and valuable, contributing to a broader discussion on nursing practice environments in complex healthcare contexts.
